# Fatal Invasive Cryptococcal Infection in an HIV-Negative Elderly Patient with Decompensated Hepatic Cirrhosis

**DOI:** 10.1155/2018/5174518

**Published:** 2018-12-31

**Authors:** Shigeru Koba, Kazuki Ueda, Masahiro Mori, Kenji Miki, Shinsaku Imashuku

**Affiliations:** ^1^Department of Internal Medicine, Uji-Tokushukai Medical Center, Uji, 611-0042, Japan; ^2^Department of Laboratory Medicine, Uji-Tokushukai Medical Center, Uji, 611-0042, Japan

## Abstract

Diagnosis of invasive cryptococcal infection in apparently nonimmunocompromised patients is difficult and often delayed. Human immunodeficiency virus- (HIV-) negative patients with decompensated hepatic cirrhosis might be at high risk of cryptococcal infection. We report here an 82-year-old Japanese female with end-stage hepatic failure and undergoing renal dialysis, hospitalized with septic shock-like symptoms. The patient had had hepatitis B virus (HBV) infection in the past. She survived only 4 days following admission. During hospitalization, she was found to have pleural effusion and ascites.* Cryptococcus neoformans* was obtained from blood culture but not from pleural effusion culture. Consequently, the patient was diagnosed as having invasive cryptococcosis in association with HBV-related hepatic cirrhosis. Unfortunately, the patient died prior to receiving antifungal agents. Twelve Japanese cases of hepatic cirrhosis-related invasive cryptococcal infection, consisting of previously described and this case, were summarized for discussion of the clinical features and outcomes.

## 1. Introduction

Invasive cryptococcal infection, or cryptococcosis, usually develops in immunocompromised hosts, e.g., in human immunodeficiency virus- (HIV-) positive patients. However, it also occurs in HIV-negative subjects. Cryptococcosis-related mortality is high and does not differ between HIV-positive and HIV-negative patients [1]. Although cryptococcal infection of the lungs and nervous system is common, gastrointestinal cryptococcosis can occur in the absence of infection of above organs [2], as innate and adaptive immune dysfunction is noted in patients with end-stage liver disease [3]. Thus, cryptococcal peritonitis has been reported, with some cases progressing to fungemia and/or meningitis in instances of alcoholic [4], hepatitis B virus (HBV) [5, 6], or hepatitis C virus (HCV) [7] related decompensated hepatic cirrhosis. We report here a fatal Japanese case of* Cryptococcus neoformans* fungemia related to HBV cirrhosis in an elderly patient with pleural effusion and ascites. We also reviewed previously reported similar cases in Japan.

## 2. Case Presentation

**Figure 1:**
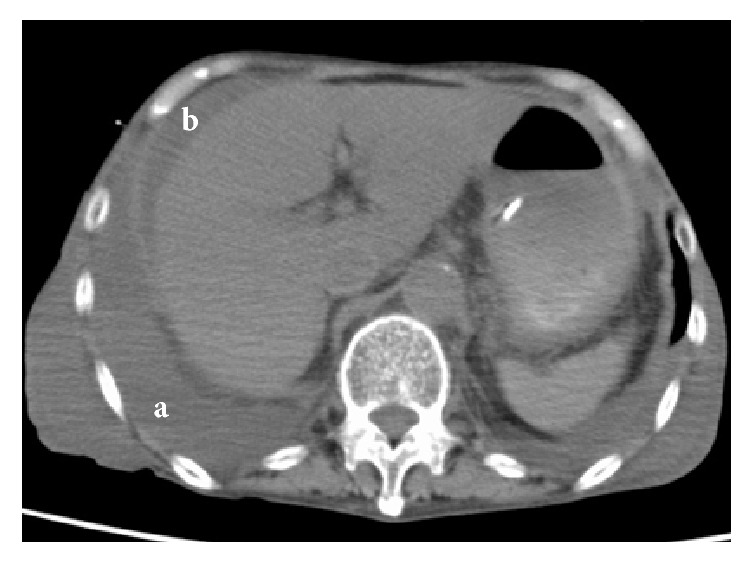
Computed tomography of the abdomen. Pleural effusion (a) and ascites (b) are seen. Within the liver, no abscess lesions were noted.

**Figure 2:**
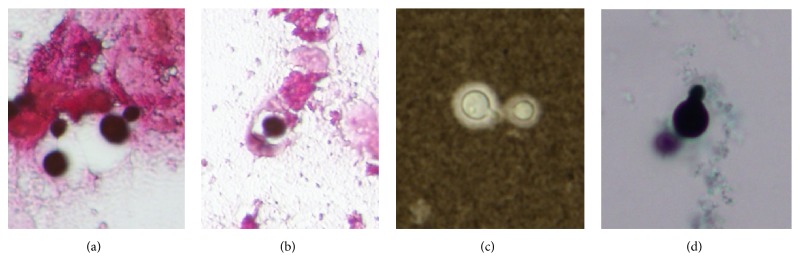
Cryptococci obtained from the blood culture. (a, b) Gram stain, (c) India ink stain, and (d) Grocott stain. The stains revealed characteristic budding-like features (a, d) and encapsulation (b, c) (original magnification, ×1000).

An 82-year-old female was transferred to the emergency department of our hospital with general malaise followed by septic shock-like symptoms. She had been undergoing renal dialysis over the past 2 years. The patient's test was negative for HIV, HCV, and human T-cell leukemia virus 1, and she did not have any type of cancer, was not undergoing chemo- or corticosteroid therapy, and showed no evidence of autoimmune diseases, but she had a history of HBV infection. She was not a drinker. Prior to admission, she had been in another hospital where she was diagnosed with end-stage hepatic failure (Child-Pugh stage C) and treated for anorexia, hypotension, and hypoglycemia. On admission, the patient complained of malaise, followed by a state of Japan Coma Scale I-3 (Glasgow Coma Scale E3V1M5), with severe hypotension (blood pressure unmeasurable). She was afebrile, severely anemic, and icteric, with liver dysfunction and hemorrhagic tendency but without peritoneal signs, such as localized guarding. Laboratory tests indicated high serum C-reactive protein levels, increased direct bilirubin, and extremely high hyaluronic acid and type IV collagen levels, in association with reduced total protein and albumin levels, as well as reduced prothrombin time and markedly low choline esterase activity. All these were compatible with decompensated liver dysfunction. For the complement system, reduced 50% hemolytic complement (CH50) and C3 levels, with normal C4 levels, were noted (Table 1). Abdominal computed tomography revealed pleural effusion and small ascites (Figure 1). Pleural effusion was aspirated; cell counts therein were determined to be 272/*μ*L, and the culture was negative. Abdominal paracentesis and spinal tap were not performed. During her admission, she was cared for by intubation in the intensive care unit because of persistent hypotension and low blood oxygen saturation (SpO_2_). Blood culture on admission yielded* Cryptococcus* (Figure 2) on day 4 of admission when she died. Also, positive serum cryptococcal antigen (titer; 1:128) was confirmed postmortem. Consequently, the patient was diagnosed as having invasive cryptococcal infection linked to HBV-related hepatic cirrhosis and died prior to receiving antifungal agents. Later, the presence of* C. neoformans* was confirmed.

*Survey of Reports of Japanese Cases. *We used the Igaku Chuo Zassi (ICHUSHI; the Japan Medical Abstract Society, www.jamas.or.jp) to survey data for patients in Japan who suffered from invasive cryptococcal infection related to liver cirrhosis and/or failure during the years 1990–2016. The keywords used were “liver cirrhosis”, “peritonitis”, “meningitis”, and “systemic cryptococcal infection”. Cases concurring with cancer were excluded. We identified 11 cases, mostly short abstracts and not full papers [8–18] (Table 2). Including the case described in the current report, a total 12 cases were analyzed. Of these cases, nine patients were over 50 years old and the male/female ratio was 9/3. The following causes of liver cirrhosis were reported: HBV (n=2), HCV (n=2), alcohol (n=2), primary biliary cirrhosis (n=2), and unknown (n=4). Treatment was known for eleven cases except for one. Seven cases received antifungal agents, of which four were alive and three had died at the time of reporting. All four cases not given antifungal agents died. Although detailed information was limited in the retrieved reports, it appears that the outcome may have been better in certain cases who were diagnosed early and had the antifungal treatment been provided in a timely manner.

## 3. Discussion

Alveolar macrophages are the first line of defense against* C. neoformans* infection, followed by neutrophils and monocytes. As a third line of defense, T- and B-cell responses are critical and produce various cytokines, such as interferon gamma and specific antibodies [19]. When such defense mechanisms break down in immunocompromised patients,* C. neoformans* emerges as an important pathogen [20].

Invasive cryptococcal infection such as fungemia and/or meningitis mostly occurs in association with immunocompromised conditions, such as acquired immunodeficiency syndrome, or with autoimmune diseases such as systemic lupus erythematosus and rheumatoid arthritis [21, 22]. On the other hand, in patients with decompensated hepatic cirrhosis,* C. neoformans* invades the host through the gastrointestinal tract and causes peritonitis even in HIV-negative patients [4–6, 20, 21]. Some of these patients have localized peritonitis, but others further develop fungemia and/or meningitis [4, 6]. Although precise mechanism remains unknown, recent reports on cirrhotic patients complicated with invasive cryptococcal infection are accumulating [8, 9, 21, 23–25].

The patient described in the current study exhibited septic shock-like symptoms on admission, with pleural effusion and ascites. Although pleural effusion as an initial clinical presentation in cryptococcosis has been reported [9, 23], the pleural fluid culture was negative in our case. During 4 days of admission with rapid deteriorating, we were unable to diagnose the disease prior to the patient's death. We missed the chance to detect cryptococcal antigen and beta-D-glucan in time [26] and failed to examine the ascites and cerebral spinal fluid, but eventually blood culture yielded* C. neoformans*. In hindsight, we speculated that the patient had already developed peritonitis and fungemia prior to referral to the hospital. This was compatible with a report that delayed diagnosis of cryptococcal peritonitis often results in death [5]. A report on a patient by Jean et al. [20] illustrates the difficulty of diagnosis, in which ascites was examined four times during 2 weeks after admission; upon fourth examination, both the ascites and serum became positive for the cryptococcal antigen. A week later, the cerebrospinal fluid culture yielded* C. neoformans*.

Including the same eight cases cited from literature, Albert-Braun et al. [4] analyzed 17 cases and Park et al. [6] analyzed 16 cases of HIV-negative cryptococcal peritonitis linked to cirrhosis. Of the 11 patients treated with amphotericin B with or without fluconazole; two patients survived [4], while 7 of 16 (44%) patients who did not receive antifungal treatment died [6]. Spec et al. reported high mortality (20/25; 80%) including 7 cases died before administration of antifungal therapy [24]. On the other hand, among the Japanese cases analyzed in the current study, antifungal agents were given to 7 of 12 (58%) patients, of which four were alive at the time of reporting. Unfortunately, the correlation of timing of antifungal agent administration and outcome could not be analyzed.

Early diagnosis of* C. neoformans* peritonitis is possible if India ink-positive encapsulated budding yeasts are visible upon microscopic examination of the ascites [5]. However, obtaining fungi from cultures of the ascites, blood, or cerebrospinal fluid takes 3–7 days. Although reports of cryptococcal peritonitis in cirrhotic patients are still limited, the incidence is likely underestimated when the physician does not recognize such a risk factor and the disease is not specifically looked for, as illustrated by the current report. When a patient presents with shock and coma, which are common clinical features of invasive cryptococcal disease [27], it appears to be already too late to offer life-saving treatment.

In suspected cases of cryptococcal fungemia, depletion of the serum complement components should be evaluated. Complement C3 as well as CD11b are essential for neutrophil swarming around* C. neoformans* [28]. The ability of the serum to mediate C3 binding to* C. neoformans* is severely impaired in patients with invasive cryptococcal infection [29]. In fact, in the case reported in the current study, reduced CH50 and C3 levels, with normal C4 levels, matched the data presented in a previous report [29]. A more recent report on a patient who received eculizumab (a monoclonal antibody that blocks complement activation) and succumbed to disseminated cryptococcosis [30] may underscore the importance of the complement system in cryptococcal infection.

In summary, to improve the outcome in patients with decompensated cirrhosis with ascites, awareness of the immune dysfunction-related risk of invasive cryptococcosis is indispensable. Early diagnosis of cryptococcal infection and prompt introduction of antifungal agents are required for life-saving. For any end-stage cirrhotic patients, beta-D-glucan and cryptococcal antigen assays as well as culture with India ink staining of blood, ascites, or spinal fluid are highly recommended.

## Figures and Tables

**Table 1:** Laboratory data on admission.

**CBC**		**Hepatic**	
WBC (3000-8500) /*µ*L	9200	CRP (0-0.29) mg/dL	9.68

Hb (11-16) g/dL	7.2	AST (13-37) U/L	58

MCV (83-100) fL	119	ALT (8-45) U/L	32

PLTs (150K-360K)/*µ*L	57K	LDH (122-228) U/L	362

**Hemostatic**		ChE (206-477) U/L	25

PT (80-100)%	26.2	g-GTP (8-33) U/L	23

PT-INR (0.9-1.1)	2.24	T. bil (0.3-1.3) mg/dL	6.68

APTT sec	60.2	D. bil (0.1-0.3) mg/dL	4.70

APTT-control sec	28.1	Total protein (6.7-8.3) g/dL	4.4

Fibg (200-400) mg/dL	88	Albumin (4.1-5.2) g/dL	1.9

FDP (0.0-2.5) *µ*g/mL	16.8	Ammonia (20-70) *µ*g/dL	173

D-dimer (0.0-1.0) *µ*g/mL	10.2	Hyaluronic acid (0-50) ng/mL	41,112

		Type IV collagen (0-140) ng/mL	872

**Renal & Electrolytes**		Ceruloplasmin (21-37) mg/dL	14.1

BUN (7.8-18.9) mg/dL	48.7	**Viral studies**	

Cre (0.45-0.82) mg/dL	3.97	HBs-Ag/HBs-Ab	neg/pos

eGFR	9	HBc-Ab	pos (59.6)

UA (2.5-5.8) mg/dL	4.1	HCV-Ab	neg

Na/ K/ Cl mmolL/L	141/ 5.2/ 105	HCV-core protein	<3

**Immunological**		HIV	neg

IgG (820-1740) mg/dL	851	HTLV-1	neg

IgA (90-400) mg/dL	424	**Pleural fluid**	

IgM (52-270) mg/dL	21	Cell counts; /*µ*L	272

C3 (80-140) mg/dL	44	Differential; mono %/ poly %	57.7/42.3

C4 (11-34) mg/dL	11.6	Protein; g/dL	2.4

CH50 (30-45) U/mL	15	ADA (40-50) U/L	17.1

Abbreviations for Table 1.

WBC=white blood cell count, Hb=hemoglobin, MCV=mean corpuscular volume, PLTs=platelet counts, PT=prothrombin time, APTT=activated partial thromboplastin time, Fibg=fibrinogen, FDP=fibrin degradation product, BUN=blood urea nitrogen, Cre=creatinine, eGFR= estimated glomerular filtration rate, UA=uric acid, Ig=immunoglobulin, CH50=50% hemolytic unit of complement, C3=complement 3, C4=complement 4, CRP=C-reactive protein, AST=aspartate aminotransferase, ALT=alanine aminotransferase, LDH=lactate dehydrogenase, ChE=choline esterase, g-GTP=gamma-glutamyl transpeptidase, T. bil=total bilirubin, D. bil=direct bilirubin, HB=hepatitis B virus, Ag=antigen, Ab=antibody, HCV=hepatitis C virus, HIV=human immunodeficiency virus, HTLV-1= Human T-cell leukemia virus type 1, ADA=adenosine deaminase.

**Table 2:** Cirrhosis-related cryptococcosis; Japanese experiences.

	Reference	Age/Gender	Causes of liver cirrhosis	Symptoms	Cryptococcal infection	Treatment	Outcome (survival)
1	Nakamura 2016	68/F	PBC	fever	peritonitis fungemia	MCFG	Died (32 days)
2	Haga 2015	59/M	HCV	dyspnea PE	pulmonary, MRSA	surgery	Died (8 days)
3	Hokari 2010	58/F	PBC	diarrhea	systemic	VCM	Died (16 days)
4	Akiyama 2010	64/M	Alcohol	seizure	meningitis	AMPH-B F-FLCZ	Died (23 days)
5	Mitomi 2004	43/M	Alcohol	headache fever, general malaise	meningitis	FLCZ AMPH-B	Died (57 days)
6	Touge 1999	60/M	HBV	headache nausea	meningitis	FLCZ	Alive
7	Arata 1996	58/M	NA	fever cavernous lung mass	pulmonary/ meningitis	FLCZ	Alive (22 days+)
8	Miyazaki 1993	70/M	HCV	disturbed consciousness	meningitis	FCLZ	Alive
9	Yoshizawa 1992	58/M	NA	fever dyspnea	fungemia systemic	antibiotics	Died
10	Okazaki 1990	43/M	NA	disturbed consciousness	systemic	NA	Died
11	Yamamoto 1990	32/M	NA	fever headache hematemesis	meningitis	5-FC MCZ	Alive
12	Present case	82/F	HBV	general malaise hypotension coma	fungemia	none	Died (4 days)

*Abbreviations.* PBC=primary biliary cirrhosis, HBV=hepatitis B virus, HCV=hepatitis C virus, NA=not available, PE=pleural effusion, MRSA= Methicillin-resistant Staphylococcus aureus, MCFG=Micafungin, VCM=Vancomycin, AMPH-B=Amphotericin-B, F-FLCZ= fosfluconazole, FLCZ= fluconazole, 5-FC= flucytosine, MCZ= miconazole.
